# Ubiquitous bacteria *Borrelia crocidurae* in Western African ticks *Ornithodoros sonrai*

**DOI:** 10.1186/s13071-015-1089-6

**Published:** 2015-09-17

**Authors:** Haitham Elbir, Aurélien FotsoFotso, Georges Diatta, Jean François Trape, Céline Arnathau, François Renaud, Patrick Durand

**Affiliations:** Aix Marseille, Université, Unité de Recherche sur les Maladies Infectieuses et Tropicales Emergentes (URMITE), UMR CNRS 7278, IRD 198, INSERM 1095, Marseille, France; MIVEGEC, UMR-CNRS 5290, IRD 224, Parasitologie-Mycologie, CHRU Site Balmès, Montpellier, France; Campus commun UCAD-IRD d’Hann, Dakar, Senegal

**Keywords:** Argasidticks, Borrelia, Multiple spacer sequence typing

## Abstract

**Background:**

In West Africa, tick-borne relapsing fever is a neglected arthropod-borne infection caused by *Borrelia crocidurae* transmitted by the argasid tick *Ornithodoros sonrai*. From an epidemiological point of view, it is of interest to know whether some genotypes of the vector are specialized in carrying certain genotypes of the pathogen.

**Findings:**

Thirty-five *O. sonrai* ticks collected in Mali, Senegal, Mauritania and Morocco confirmed to be *B. crocidurae*-infected*,* were genotyped by 16S rRNA gene sequencing. *B. crocidurae* was genotyped by Multispacer Sequence Typing. The 35 *O. sonrai* ticks grouped into 12 genotypes with strong geographical structuration. MST resolved the 35 *B. crocidurae* isolates into 29 genotypes with pairwise divergence of 0.09 - 1.56 % without strict geographical structuration as genotype ST22 was found in Mali, Senegal and Mauritania. There was no evidence of tick-borrelia specialization as one *O. sonrai* genotype carried several *B. crocidurae* genotypes and one *B. crocidurae* genotype was found in different *O. sonrai* genotypes.

**Conclusions:**

This report illustrates a non-specialized circulation of *B. crocidurae* borreliae within *O. sonrai* ticks in West Africa.

## Background

*Borrelia crocidurae* is one of the spirochetes responsible for tick-borne relapsing fever in North and West Africa [1]. In these countries, borreliae are maintained between *Ornithodoros sonrai* argasid ticks and rodents while the humans get infected accidently [2, 3]. Accordingly, *B. crocidurae* has been detected in *O. sonrai* ticks collected in Tunisia, Morocco, Mauritania, Senegal [4] and Mali [3]. In Morocco, borreliae infection was found in 10.2 % of ticks and 8.6 % of tested rodents and insectivores [5]. In Mali, *B. crocidurae* was found in 17.3 % of *O. sonrai* ticks and its diversity was established and characterized in 15 samples by multi-locus sequence typing (MLST) [3]. In patients, an average incidence of 11 per 100 person-years was reported in humans at Senegal [2].

It has already been reported that multiple strains of *B. crocidurae* were probably transmitted by ticks collected from the same geographical area and their progeny [6]. This observation however, was made before tools were available for a fine discrimination between both ticks and borreliae groups. Recently, such sequencing-based tools allowed refining the diversity of *O. sonrai* ticks into nine subgroups [7]. Likewise, MLST [3] and multiple spacer sequence typing (MST) [8] discriminated groups among *B. crocidurae*. Genotyping found a strong geographical structuration of the *O. sonrai* tick population [7], however, it remains unknown whether certain *O. sonrai* genetic variants are specialized in certain genetic variants of *B. crocidurae*. The aim of the present work was to investigate this question using modern molecular tools for genotyping *O. sonrai* ticks infected by *B. crocidurae*.

## Findings

The National Ethics Committee of Senegal approved the protocol used for human samples. The study protocol was also approved by the Steering Committee of the Institut de Recherche pour le Développement (IRD) Special Programme Evolution Climatique et Santé (IRD, Montpellier, France), reference project ATI-ECS-07-H/2002.

*O. sonrai* ticks here studied are not registered as endangered species. Ticks were collected using a flexible tube and a portable aspirator, inside houses and private lands after the owner of the house and land gave permission to conduct the study on the site. Collected ticks were immediately preserved in ethanol 70 %, before total DNA (tick and *Borrelia*) was extracted and purified by using the DNeasy Tissue extraction kit (Qiagen, Hilden, Germany) according to the manufacturer’s instructions and stored at −20 °C until used as a template for the PCR-based amplification of tick or *Borrelia* genes.

The presence of *B. crocidurae* was confirmed in ticks by flagellin gene PCR-sequencing as previously described [4]. Genotyping of *B. crocidurae* was then done by using MST as previously [8]. The nucleotide sequences were edited using ChromasPro software (www.technelysium.com.au/chromas.html). Similarities between MST types were determined after multiple alignments using the Muscle software implemented in MEGA5 package [9]. The five-spacer sequences analyzed herein were concatenated. Relationships among *B. crocidurae* genotypes detected in ticks plus previously published 7 spacer types (STs) of human *B. crocidurae* isolates [8] were inferred using the Bayesian phylogenetic analyses in MrBayes v3.1.2 using default parameters [10]. Each particular combination of the five spacer sequences was assigned to a spacer type (ST) number.

In parallel, tick genotyping was done by 16S rRNA gene sequencing as previously described [7]. Modeltest 3.4 [11] was used to select the appropriate model of molecular evolution. The best-fitting ML model was HKY85 [12] for 16S rRNA sequences. The highest-likelihood DNA and corresponding bootstrap support values were obtained by PhyML (freely available at http://mobyle.pasteur.fr/cgi-bin/portal.py#welcome) using NNI (Nearest Neighbor Interchange) branch swapping and 1000 bootstrap replicates.

A total of 35 ticks - *Borrelia crocidurae* complexes collected in four countries (14 sites) and 13 infected patients were used in this study. All the new spacer nucleotide sequences of *Borrelia crocidurae* have been deposited in the GenBank database under accession numbers KF843734-KF843765 and described previously, including sequences of *B. crocidurae* from patients (JQ398819-JQ398841) [1]. The 16S rRNA sequences of *Ornithodoros sonrai* were also deposited in GenBank KP644211-KP644222).

## Results

In all PCR-based experiments, negative controls remained negative. The *B. crocidurae*-infected ticks here investigated comprised one tick from Morocco (1 site), three ticks from Mauritania (1 site), twenty-seven ticks from Senegal (8 sites) and four ticks from Mali (4 sites) (Table 1).

**Table 1:** Geographical sites and 16 s rRNA genotypes for *Ornithodoros sonrai* ticks used in this study from Trape and colleagues [7]

Country	Location	Coordinates	Code of ticks	Code of 16S rRNA group of ticks
Morocco	Boudnib	31°59’N-03°59’W	BOUD1310	Os01
Mauritania	Amridjiel (Oum El Khez)	17°00’N-10°59’W	OUM1047	Os06
			OUM1044	Os05
			OUM 1063	Os01
Senegal	Dielmo	13°43’N-16°24’W	DL1646 DL1650	Os11
			DL1669 DL1675	
			DL1676 DL1706	
			DL1657 DL1661	Os12
			DL1662 DL1674	
			DL1677 DL1680	
			DL1709	
	Thianène (Darou Mousti)	15°04’N-16°00’W	DM129 DM136	Os03
			DM148 DM150	
			DM155 DM160	
	Néma-Nding	13°42’N-16°25’W	NNT919	Os10
	Keur Aliou Gueye	13°47’N-16°24’W	KAG349 KAG351	Os12
	Kanène Khar	15°30’N-16°01’W	KKH113	Os03
	Keur Lamine Socé	13°44’N-16°25’W	KLS363 KLS364	Os12
	Kéniéba	14°05’N-12°03’W	KNB305	Os07
	Taouey (Richard-Toll)	16°27’N-15°42’W	RT12	Os02
Mali	Makania (Diamou)	14°04’N-11°09’W	DIA999	Os03
	Dianguirdé (Diéma)	14°30’N-09°01’W	DIEM1207	Os04
	Kolomina (Doubala)	14°28’N-08°01’W	DOUB1237	Os09
	Sogoli (Ké-Macina)	13°58’N-05°28’W	KEM1410	Os08

**Fig. 1:**
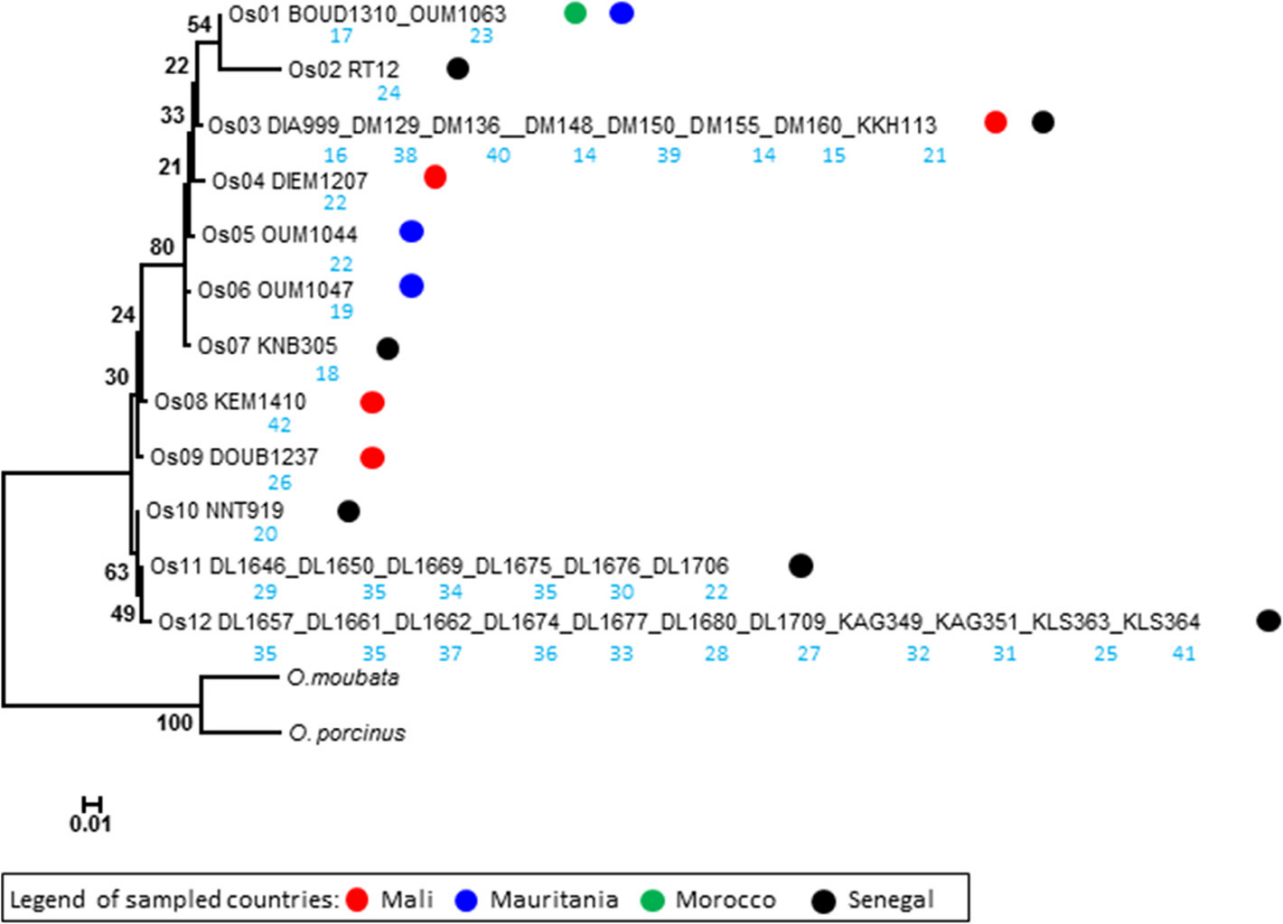
Phylogram (PhyML 1000 bootstraps) of partial 16S rRNA gene sequence data (441 nucleotides) of *Ornithodoros sonrai* sequences (35 sequences were grouped in 12 Os). Bootstrap values are shown in black (scale bar, 0.01 substitutions per site). Two 16S rRNA gene sequences of *Ornithodoros moubata* (GenBank accession number AB073679) and *Ornithodoros porcinus* (GenBank accession number AB105451), were treated as outgroups. Os: code of 16S rRNA groups. Number in blue: Spacer-Type of *Borrelia crocidurae*

As for tick genotyping, all positions containing gaps and missing data were eliminated from the 441-bp aligned fragment of 16S rRNA gene sequences of *O. sonrai*. There was a total of 435 positions with 36 variable sites and 20 parsimony-informative sites in the final dataset. The 35 *O. sonrai* ticks used for the genetic analysis yielded 12 different genotypes (Table 1, Fig. 1). All ticks in the same clade (i.e. Os) had identical 16S rRNA sequences (Fig. 1).

One tick (Boud1310) collected in Morocco yielded one genotype (Os01) common with a tick (Oum1063) from Mauritania, the two other ticks collected in Mauritania yielded two genotypes. The twenty-seven ticks collected in Senegal (8 sites) yielded six genotypes and the four ticks collected in Mali yielded four genotypes (Fig. 1). More specifically, the Os04, Os09 and Os10 genotypes were found only in Mali whereas the Os03 genotype was found both in Mali and Senegal; the Os05 and Os06 genotypes were found only in Mauritania whereas the genotype Os01 was found both in Morocco and Mauritania; the genotypes Os02, Os07, Os10, Os11, Os12 were found only in Senegal (Fig. 1).

**Fig. 2:**
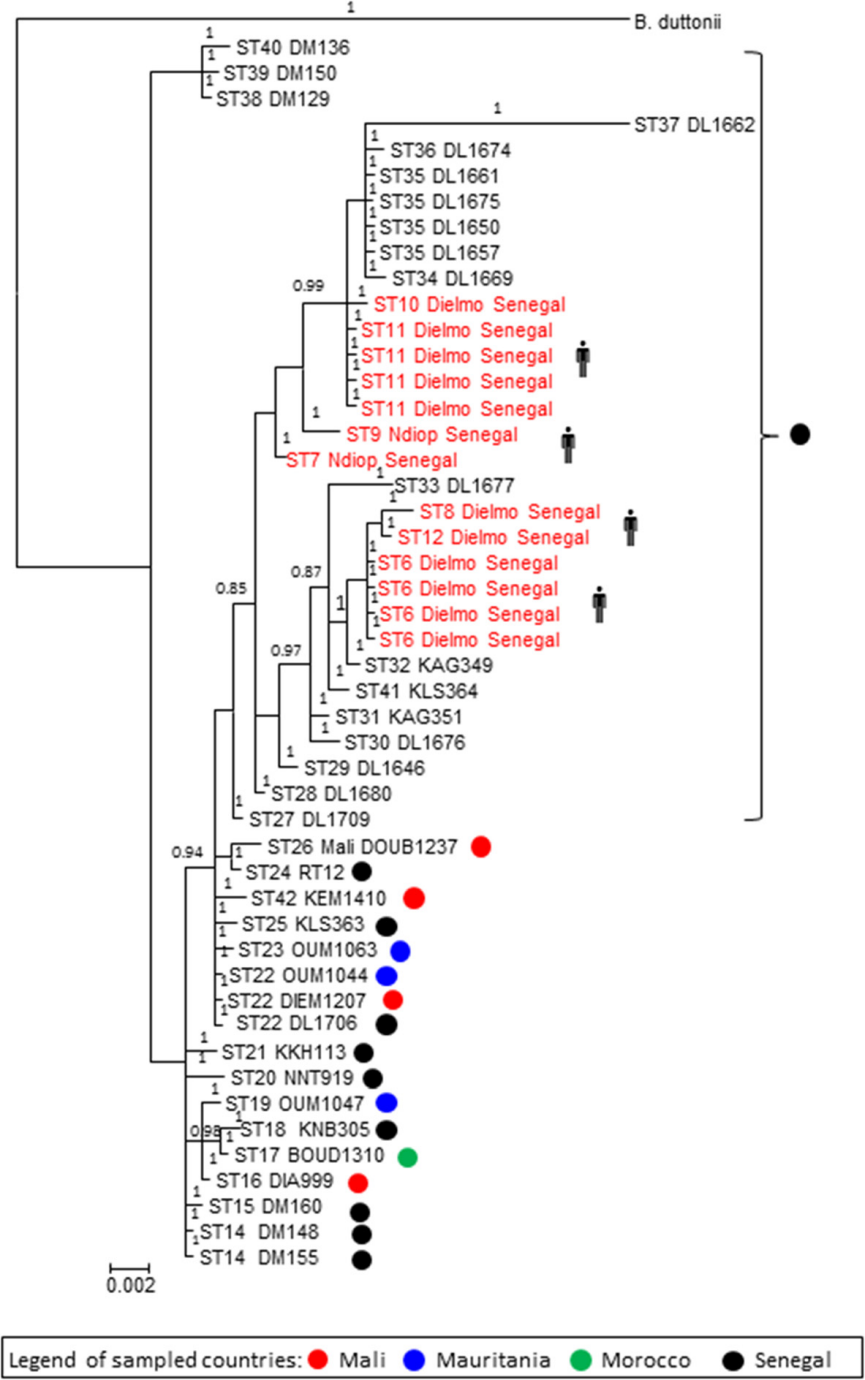
Bayesian MCMC analysis based on five intergenic spacers sequences for 48 *Borrelia crocidurae* strains in *Ornithodoros sonrai* ticks and human samples. The site of Dielmo (Senegal) is only indicated in the figure for comparison between STs *B. crocidurae* found in humans and ticks. Numbers represent posterior probabilities. Red colour indicates *Borrelia* from humans ( symbol) and black colour indicates *Borrelia* from ticks

As for *B. crocidurae* genotyping, the analysis of the spacer sequences derived from the 35 *B. crocidurae* isolates from ticks yielded 29 spacer types (STs) named ST14–ST42 and 13 *B. crocidurae* isolates from patients yielded 7 spacer types (STs) previously named ST6–ST12 [8] (Fig. 2). Pairwise divergence between *B. crocidurae* ranged from 0.09 to 1.56 %. ST22 was detected in ticks collected in Mali, Senegal and Mauritania. The ST35 in Dielmo (Senegal) was found in tick genotype Os11 and Os12 and five STs (STs 14, 15, 38, 39 and 40) were found in Darou Mousti (Senegal). Three STs named ST38–ST40 were found in Senegal and carried by genotype Os03 (Figs. 1 and 2). Tick genotypes Os01, Os03, Os11 and Os12 carried more than one genotype of *B. crocidurae.* The phylogenetic tree constructed from 35 spacer sequences of *B. crocidurae* separated the strains into two clades. A first clade comprised of 32 *B. crocidurae* genotypes and a second clade comprised of three *B. crocidurae* genotypes (STs 38–40). A phylogenetic tree combining the STs found here in ticks, with the STs previously reported in patients [8], yielded a geographical clustering of *B. crocidurae* in patients and ticks from Dielmo, Senegal (Fig. 2).

## Discussion

The presence of several genetic variants of *B. crocidurae* has recently been reported in Mali [3]. In Mali, plasmidogram found six profiles among 15 isolates which yielded four MLST groups after sequencing the 16S rRNA, *flaB*and *glpQ* genes and the IGS [3]. Here, we further observed a high diversity of *B. crocidurae* in West Africa, in observing 27 genetic variants determined by MST, among 35 *B. crocidurae* samples. While plasmidogram and MLST required cultured borreliae [3], we applied MST directly to infected ticks as we previously did on the blood collected from infected patients in Senegal [1, 8].

Previous study showed differences in the infection rate of *O. sonrai* from different regions, compatible with differences in vector competence [4]. Our study does not verify this hypothesis. Indeed, the diversity of tick-borne *B. crocidurae* was not superimposed on that of *O. sonrai*. We found that one *B. crocidurae* ST was carried by different genotypes of *O. sonrai*. In addition, one *O. sonrai* genotype was carrying different *B. crocidurae* STs. Accordingly, we observed that one *B. crocidurae* ST22 was circulating in Mali, Mauritania and Senegal with different tick genotypes (Os04, Os05 and Os11). The ST35 (4 bacterial samples) was also circulating in two tick genotypes (Os11, Os12). Figure 2 also shows the *B. crocidurae* ST14 detected in two ticks of one sampled site (Darou Mousti, Senegal). These findings indicate that, within *O. sonrai*, there is no specialization of certain tick genetic variants for their capacity to host *B. crocidurae*.

Also, we were able to compare *B. crocidurae* MST genotypes in patients and ticks of the same village of Dielmo, rural Senegal. In this village, the *B. crocidurae* genotypes previously reported in patients unsurprisingly form a subset of the strains circulating in the tick vectors, confirming *O. sonrai* as the vector of *B. crocidurae* in this village.

This analysis indicates that MST is well adapted to study the diversity of borreliae on a small, kilometer geographical size; other tools may be more suitable on a larger scale.
